# Fifteen-Centimeter Giant Tamoxifen-Associated Endometrial Polyp Presenting With Constipation: A Case Report and Review of the Literature

**DOI:** 10.1155/2024/9826447

**Published:** 2024-07-08

**Authors:** Sompon Apornvirat, Komsun Suwannarurk

**Affiliations:** ^1^ Department of Pathology Thammasat University Hospital, Pathum Thani, Thailand; ^2^ Chulabhorn International College of Medicine Thammasat University, Pathum Thani, Thailand; ^3^ Department of Obstetrics and Gynecology Faculty of Medicine Thammasat University, Pathum Thani, Thailand

**Keywords:** constipation, endometrial polyp, pressure symptom, tamoxifen

## Abstract

Endometrial polyps are benign disorganized growth of endometrial glands and stroma in the uterine cavity. They are associated with subfertility, abnormal uterine bleeding, and tamoxifen use. While most polyps are smaller than 2 cm in size, rare giant polyps can cause concerns over malignancy. We report a case of a 15 cm giant endometrial polyp in a 58-year-old woman with a history of tamoxifen use who presented with an uncommon complaint of constipation. Additionally, a literature review of giant endometrial polyp cases is presented. This case represents the largest reported endometrial polyp to date.

## 1. Introduction

Endometrial polyps are benign disorganized growth of endometrial glands and stroma in the uterine cavity. They are associated with subfertility, abnormal uterine bleeding, and tamoxifen use, while their pathogenesis is still not fully understood [1–4]. The majority of endometrial polyps are less than 2 cm in size [5]. Larger polyps can cause concerns over malignancy due to their size and associated symptoms. This may lead to overdiagnosis of malignancy or unnecessary treatment. Herein, we report a rare case of a giant tamoxifen-associated endometrial polyp in a woman who presented with constipation.

## 2. Case Presentation

A 58-year-old female, G3P2A1, with a history of hypertension, uterine leiomyoma, and breast cancer, presented with increasing constipation for 5 months. She had reached menopause 9 years before this visit and denied any other hormonal drug use except tamoxifen.

Nine years prior to this visit, she had menometrorrhagia and a suprapubic mass, approximated to a 16-week size uterus, was found. She was diagnosed with leiomyoma by ultrasonography and achieved menopause soon after with only symptomatic treatments.

Four years before presentation, she found a mass in her left breast. A mammogram with ultrasound revealed a BI-RADS 5 spiculated hypoechoic nodule with hypervascularity and nonparallel orientation. A core needle biopsy confirmed the diagnosis of invasive ductal carcinoma. She underwent total mastectomy with sentinel lymph node biopsy, and her final diagnosis was a hormone-positive invasive ductal carcinoma of no special type Grade 2 and Stage 1, pT1N0M0. Adjuvant chemotherapy with doxorubicin and cyclophosphamide was given, followed by tamoxifen 20 mg per day. No cancer recurrence was detected.

**Figure 1:**
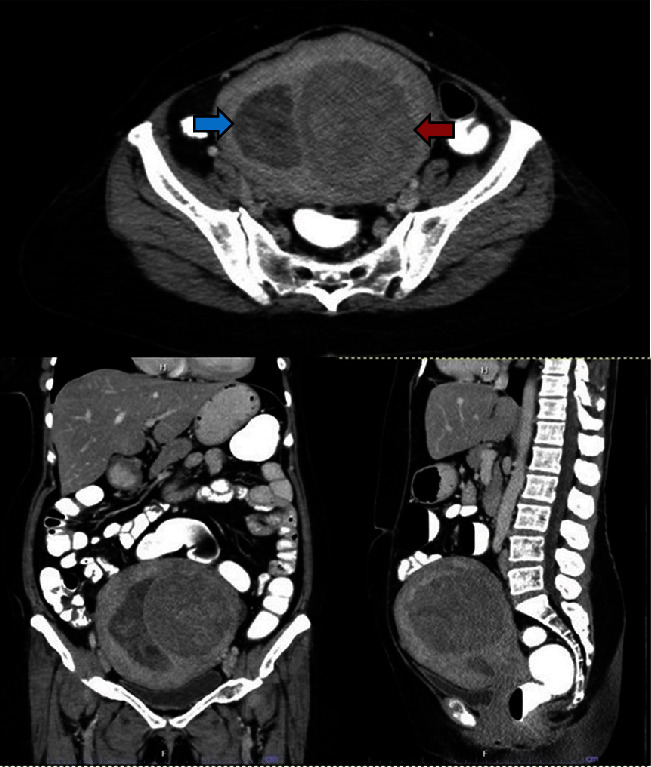
Abdominal CT scan demonstrated enlarged uterus with well-defined heterogenous enhancing lesions at the left side of uterine wall (red arrow). Heterogenous hypodense lesion in the right side of the uterine cavity with 3.8 cm thickness (blue arrow).

At the gynecological clinic, she complained of increasing constipation for 5 months and denied other symptoms. A large painless abdominal mass was palpated during physical examination, approximated to an 18-week size uterus. Transabdominal ultrasound revealed 6.0 cm endometrial thickness and 8.0 cm submucosal mass at the posterior uterine wall. Endometrial biopsy yielded an unsatisfactory result with only blood clots and a few tiny fragments of atrophic endometrium. She underwent abdominal computed tomography, which demonstrated an enlarged uterus with a 3.8-cm-thick heterogeneous hypodense lesion in the uterine cavity and a well-defined heterogenous enhancing lesion in the left uterine wall (Figure 1). The provisional diagnosis was an unspecified endometrial mass with submucosal leiomyoma. After thorough counseling with the patient, we agreed on transabdominal hysterectomy with bilateral salpingo-oophorectomy and complete surgical staging due to the uncertain nature of the uterine mass.

**Figure 2:**
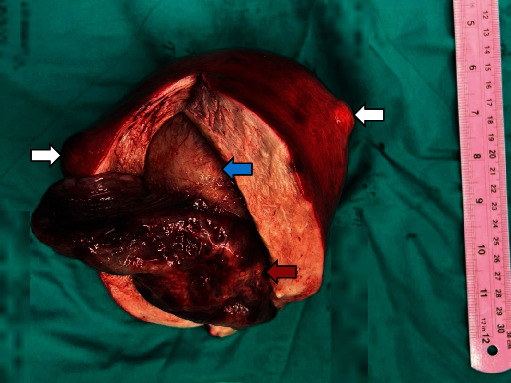
Macroscopic image of the uterus showing the endometrial polyp (red arrow) and the underlying submucosal leiomyoma (blue arrow). Two smaller subserosal myomas can also be seen in this image (white arrows).

On gross examination, the globular-shaped uterus contained a 15.0 × 6.5 × 4.8 cm mass in the endometrial cavity (Figure 2). This lesion had pale brown gelatinous to cystic cut surfaces. There was an 11-cm submucosal mass of likely leiomyoma beneath the endometrial lesion. Two smaller subserosal masses were identified with similar gross features to the submucosal lesion. The left ovary, left fallopian tube, omentum, and pelvic lymph nodes were grossly unremarkable. The right ovary and fallopian tube were not identified.

**Figure 3:**
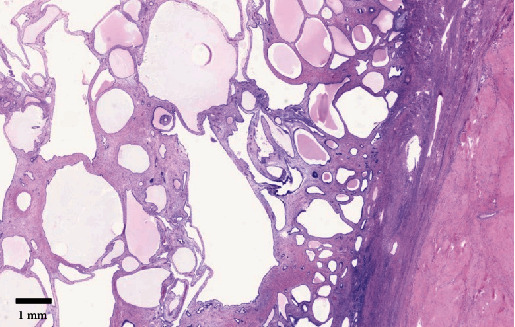
H&E stain 7.2x magnification image showing cystic endometrial polyp with the underlying leiomyoma.

**Figure 4:**
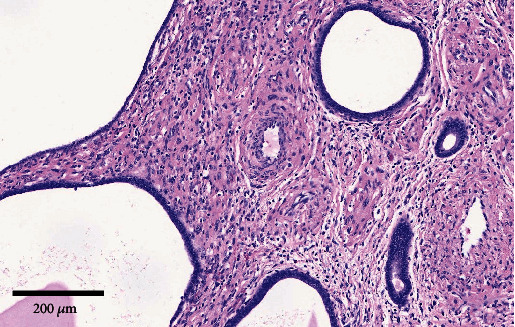
H&E stain 100x magnification image of the endometrial polyp showing flattened epithelial cells with thick-walled blood vessels in the stroma.

Microscopic examination of the endometrial polyp revealed dilated endometrial glands with attenuated epithelial cells (Figure 3). The stroma was denser than normal endometrial stroma and contained thick-walled blood vessels (Figure 4). The underlying submucosal and subserosal lesions were leiomyomas with prominent hyalinization. The left ovary was atrophic, and the left fallopian tube was unremarkable. No malignancy was detected in the omentum, peritoneal washing fluid, and pelvic lymph nodes.

After the surgery, the patient recovered well. Her symptoms improved without any surgical complications. After a year of follow-up, her pelvic examination showed no sign of recurrence.

## 3. Discussion

Tamoxifen is a selective estrogen receptor modulator (SERM) commonly used to treat breast cancer. It has estrogenic effects on the endometrium and is associated with several uterine pathologies, including endometrial polyps, endometrial hyperplasia, endometrial carcinomas, and uterine sarcomas [6, 7].

Endometrial polyp is the most common endometrial lesion associated with postmenopausal tamoxifen use [7]. In this setting, polyps are usually larger than polyps in postmenopausal hormonal replacement therapy patients and unexposed postmenopausal patients [8]. Microscopically, tamoxifen-associated polyps have more prominent stromal fibrosis, a higher chance of mucinous metaplasia, and, most importantly, a higher chance of containing malignant tumors [7, 8].

One of the major concerns regarding endometrial polyps is the presence of malignancy. A meta-analysis by Sasaki et al. [5] identified abnormal uterine bleeding, menopausal status, age > 60 years, diabetes mellitus, systemic arterial hypertension, obesity, and tamoxifen use as risk factors. History of breast cancer, hormonal therapy, parity, and size of the polyps was not associated with malignancy in this meta-analysis. Vitale et al. [9] recommended removal of large polyps of more than 2 cm in postmenopausal women or polyps in patients with cancer risk factors. They also suggested managing malignant polyps as per oncologic criteria.

Rare endometrial polyps that exceed 4 cm in size have been termed giant endometrial polyps. We reviewed case reports of these polyps in English literature and summarized important data in Table 1. The largest polyp reported was 12 cm in length. The most common presenting symptom was postmenopausal bleeding. Five out of 20 cases had history of hormonal use, and four cases had associated malignancy [10–26].

This patient presented with constipation. While leiomyoma can be a rare cause of constipation [27], we believe that the mass effect from this polyp also contributed to the symptom. She had a few known cancer risk factors, including postmenopausal status, hypertension, and a history of tamoxifen use. Due to the sheer size of the mass with questionable malignancy status, transabdominal hysterectomy was a treatment of choice, and additional specimen acquisition was necessary for evaluation.

## 4. Conclusion

Giant endometrial polyps are rare, and their pathogenesis is not fully understood. Evaluation for risks of malignancy should be performed when dealing with such cases. In this paper, we reviewed other giant polyps that were reported in the literature and present, to the best of our knowledge, the largest case of endometrial polyps that is reported in detail.

## Figures and Tables

**Table 1:** Clinical characters from previous reports.

	**Year**	**Age (year)**	**Symptom**	**Hormone**	**Size (cm)**	**CP**
Nomikos, Elemenoglou, and Papatheophanis [10]	1998	74	AUB	TAM	8	CH
Erdemoglu et al. [11]	2008	55	Mass	TAM	10 × 6 × 3	
Kutuk and Goksedef[12]	2011	63	AUB	RALOX	5.2 × 4.5 × 4.0	
Çil et al. [13]	2013	83	AUB		8 × 4 × 3	
Narin et al. [14]	2013	66	Pain/AUB		12 × 6 × 5	
Unal et al. [15]	2014	70	Pain	Phyto	10 × 9.5 × 7	
Meena et al. [16]	2017	65	AS		8.5 × 1.5	
Tyagi et al. [17]	2017	48	AUB		9 × 3 × 2.5	
Tyagi et al. [17]	2017	65	AS		8.5 × 1.5	
Tyagi et al. [17]	2017	60	AUB		8 × 4	
Sharma [18]	2018	58	AUB		5 × 4 × 1	
Alnimer, Zaghmout, and Azher [19]	2018	57	AUB		4	EC/CS
Temtanakitpaisan, Kuo, and Huang [20]	2018	38	Mass		12 × 0.5	
Sato et al. [21]	2018	76	AUB		7 × 5 × 3	ESS
Staalduinen et al. [22]	2018	70	AUB		5.3	
Staalduinen et al. [22]	2018	66	AUB		8	
Öztürk et al. [23]	2019	53	Pain	TAM	5 × 2.5 × 1.5	ASC
Welp, Temkin, Sullivan [24]	2020	67	AUB		7	SC
Nair et al. [25]	2021	67	AUB		7 × 4 × 4	
Silverwood et al. [26]	2022	69	AUB		4.2	SC

Abbreviations: AS: asymptomatic, ASC: atypical stromal cells, AUB: abnormal uterine bleeding, CH: complex hyperplasia with cellular atypia, CP: complicating pathology in the polyp, EC/CS: endometrioid carcinoma with carcinosarcoma component, ESS: low- and high-grade endometrial stromal sarcoma, Hormone: associated hormonal use, Phyto: phytoestrogen (thyme tea), RALOX: raloxifene, SC: serous carcinoma, Symptom: presenting symptom, TAM: tamoxifen.

## Data Availability

Four additional hematoxylin and eosin slides were digitally scanned and are available upon request.
